# Humanized Mouse as a Tool to Predict Immunotoxicity of Human Biologics

**DOI:** 10.3389/fimmu.2020.553362

**Published:** 2020-10-15

**Authors:** Kylie Su Mei Yong, Zhisheng Her, Sue Yee Tan, Wilson Wei Sheng Tan, Min Liu, Fritz Lai, Shi Min Heng, Yong Fan, Kenneth Tou En Chang, Cheng-I Wang, Jerry Kok Yen Chan, Jianzhu Chen, Qingfeng Chen

**Affiliations:** ^1^ Institute of Molecular and Cell Biology, Agency for Science, Technology and Research (A*STAR), Singapore, Singapore; ^2^ Key Laboratory for Major Obstetric Diseases of Guangdong Province, The Third Affiliated Hospital of Guangzhou Medical University, Guangzhou, China; ^3^ Department of Pathology and Laboratory Medicine, KK Women’s and Children’s Hospital, Singapore, Singapore; ^4^ Department of Pathology, Duke-NUS Graduate Medical School, Singapore, Singapore; ^5^ Singapore Immunology Network, Agency for Science, Technology and Research (A*STAR), Singapore, Singapore; ^6^ Department of Reproductive Medicine, KK Women’s and Children’s Hospital, Singapore, Singapore; ^7^ Experimental Fetal Medicine Group, Yong Loo Lin School of Medicine, National University of Singapore, Singapore, Singapore; ^8^ Interdisciplinary Research Group in Infectious Diseases, Singapore-Massachusetts Institute of Technology Alliance for Research and Technology, Singapore, Singapore; ^9^ The Koch Institute for Integrative Cancer Research and Department of Biology, Massachusetts Institute of Technology, Cambridge, MA, United States; ^10^ Department of Microbiology and Immunology, Yong Loo Lin School of Medicine, National University of Singapore, Singapore, Singapore

**Keywords:** cytokine storm, biologic testing, humanized mice, immunotoxicity, inflammation

## Abstract

Advancements in science enable researchers to constantly innovate and create novel biologics. However, the use of non-human animal models during the development of biologics impedes identification of precise *in vivo* interactions between the human immune system and treatments. Due to lack of this understanding, adverse effects are frequently observed in healthy volunteers and patients exposed to potential biologics during clinical trials. In this study, we evaluated and compared the effects of known immunotoxic biologics, Proleukin^®^/IL-2 and OKT3 in humanized mice (reconstituted with human fetal cells) to published clinical outcomes. We demonstrated that humanized mice were able to recapitulate *in vivo* pathological changes and human-specific immune responses, such as elevated cytokine levels and modulated lymphocytes and myeloid subsets. Given the high similarities of immunological side effects observed between humanized mice and clinical studies, this model could be used to assess immunotoxicity of biologics at a pre-clinical stage, without placing research participants and/or patients at risk.

## Introduction

The pharmaceutical industry is constantly developing new biologics; however, accurate pre-clinical evaluation of human immune responses to biologics is an existing challenge (1, 2). The immune system is a complex biological system which can be affected by biologics that have an ability to alter immune-mediated activities and are major immunotoxic risk factors to humans (3, 4). Even though many aspects of the human immune system are found to be species-specific (5) and most antibody-based biologics are designed for human targets (6), non-human animal models are often used to evaluate if biologics may cause immunological toxicity at a pre-clinical stage (7, 8). Due to evolutionary divergence in the immune systems between humans and animals, it is common that pre-clinical studies fail to identify exact immunological side effects of biologics, making it difficult to extrapolate conclusions from experimental data, therefore slowing or halting the translation of treatments (9, 10). For example, cell expression marker CD28 is only expressed on 80% of CD4^+^ and 50% of CD8^+^ T cells in humans but on 100% of CD4^+^ and CD8^+^ T cells in mice (11). In addition to this, human-specific CD3, upregulation of HLA-DR on activated T cells, production of IL-8 and Toll-like receptor 10 (TLR10) are not found in non-humanized mice (12). Due to these differences, it is common that animal models are refractory to an array of biologics that are human-specific. Therefore, to expand the existing range of treatments available and minimize the test of biologics with severe immunological side effects on humans, it is paramount to utilize animal models with high human-specificity (13, 14). The aim of this study is to investigate if mice reconstituted with a human immune system can be used as a platform for screening immunotoxicity and recapitulating mechanisms of the human immune responses to biologics such as, Proleukin^®^/IL-2 and OKT3.

An example of a biologic that causes severe immunological side effects in clinical patients is Proleukin^®^/IL-2. Proleukin^®^/IL-2 is a form of recombinant IL-2 used for the treatment of cancers such as, malignant melanoma and metastatic renal cell carcinoma (15, 16). IL-2 mediated toxicity is triggered by the direct binding of IL-2 to endothelial cells expressing IL-2 (17) and *via* the stimulation of IL-2 receptor-positive effector immune cells which releases vasoactive factors (18–20). IL-2 has a great propensity to induce adverse effects which includes “cytokine storm”, capillary leak syndrome and breathing difficulties (21, 22) which limits the widespread use of Proleukin^®^/IL-2 therapy in clinics. These toxic effects require some patients to turn to alternative therapies including the use of IL-2 variants (17, 23, 24) that circumvent CD25 binding or completely withdraw from therapy after a limited number of treatment cycles even though Proleukin^®^/IL-2 is capable of inducing long-term clinical remission at a low cost (25, 26).

Another biologic known to trigger severe immunological side effects in clinics is OKT3. Utilizing hybridoma technology, OKT3 was engineered to target CD3 receptor, a membrane protein on the surface of circulating T cells (27, 28). During the initial stage, OKT3 activates T cells but subsequently promotes activated T cells to undergo apoptosis (29, 30). Due to the activation of T cells, a common side effect observed post-administration of OKT3 is the induction of a cytokine storm, which results in systemic release of inflammatory cytokines, predominantly interferon-*γ* (IFN-*γ*) and tumor necrosis factor alpha (TNF-α), which may cause a variety of adverse clinical conditions including, organ failure and pulmonary edema (31–33). Even though pre-clinical tests were conducted *in vitro* on human lymphocytes and *in vivo* on animal models, these studies failed to precisely identify the full spectrum of immunological side effects caused by a range of biologics (34–39).

## Methods

### Fetal Liver Processing and Cell Isolation

Human fetal liver (FL) samples, male and female, 16–23 weeks of age were obtained from Kandang Kerbau Women’s and Children’s Hospital (KKH) with informed and written consent from patients. SingHealth and National Health Care Group Research Ethics Committees Singapore specifically approved this study (CIRB Ref: 2012/064/B), and all experimental procedures were conducted in accordance to the protocol. FLs were processed and digested with collagenase VI (2 mg/ml in Dulbecco’s modified Eagle’s medium (DMEM)) (Thermo Fisher Scientific, USA) for 15 min at 37°C with constant rotation. Digested tissue was passed through a 100 µm mesh to obtain single-cell suspension and isolated for human CD34^+^ cells with a CD34-positive selection kit (STEMCELL Technologies, USA), according to the manufacturer’s instructions. The purity of the CD34^+^ cells was 90–99% as determined by flow cytometry.

### Mice

NOD-*SCID* *IL2Rγ^−/−^* (NSG) mice (Stock #005557) were obtained from the Jackson Laboratory and bred in the animal facility at A*STAR, Biological Resource Centre (BRC). Neonate mice were sub-lethally irradiated (100 rads) within 72 h of birth and infused with human CD34^+^ fetal liver hematopoietic stem/progenitor cells (HSPCs) *via* intra-hepatic injection [96]. At 12-weeks post-transplantation, flow cytometry was used to determine human immune cell reconstitution levels in the peripheral blood of mice. A random mix of 13–15-week-old male and female mice were used in this study. Different donors were used for Proleukin^®^/IL-2 (n = 3) and OKT3 (n = 3) experiments. The International Animal Care and Use Committee (IACUC), A*STAR specifically approved this study with the protocol number (BRC #151034). All animal experimental procedures were conducted in accordance to the protocol.

### Proleukin^®^/IL-2 and OKT3 Treatment

Proleukin^®^/IL-2 (Aldesleukin, Prometheus Laboratories Inc., USA) and OKT3 (Biolegend, USA) were procured. Dosage as per clinical scenarios, humanized mice were administered intravenously (IV) with either saline (n = 5), Proleukin^®^/IL-2 (600,000 IU) once daily for 5 days (n = 10) or a single dose of OKT3 (1 mg) injected only once (n = 9).

### Sample Preparation for Flow Cytometry

To detect intracellular cytokines and chemokines by flow cytometry, mice administered with Proleukin^®^/IL-2 and saline-treated control groups of the same experiment were injected with 0.25 mg of BFA (Sigma-Aldrich, USA) at the endpoint of the experiment (144 h) and sacrificed 6 h later. Submandibular blood collection was carried out in EDTA tubes (Greiner Bio-One, Austria), and red blood cells (RBCs) were lysed using RBC lysis buffer (Life Technologies, USA) prior to flow cytometry staining. Spleen and lymph nodes were digested in a mixture of collagenase IV (GIBCO, UK), DNase I (Sigma Aldrich, USA) and meshed through a 70 µm filter (Thermo Fisher Scientific, USA) in DMEM medium (Thermo Fisher Scientific, USA). When necessary, cell suspensions were subjected to red blood cell lysis (GIBCO, UK). The single-cell suspension was washed and re-suspended in media supplemented with 10% fetal bovine serum (FBS) (Thermo Fisher Scientific, USA).

### Flow Cytometry

Single cell suspensions from spleen or blood (PBMCs) were stained with LIVE/DEAD fixable blue dead cell stain kit (Life Technologies, USA) for 30 min at 4°C and washed in PBS containing 0.2% BSA and 0.05% sodium azide. For staining of surface markers, the cells were incubated first with primary antibodies, CD45.1 (A20; Biolegend, USA), CD3 (UCHT1; BD Biosciences, USA), CD4 (RPA-T4; BD Biosciences, USA), CD8 (SK1; Biolegend, USA), CD11c (3.9; Biolegend, USA), CD16 (3G8; BD Biosciences, USA), CD19 (HIB19; Biolegend, USA), CD25 (2A3; BD Biosciences, USA), CD39 (TU66; BD Biosciences, USA), CD45 (HI30; BD Biosciences, USA), CD45RA (HI100; BD Biosciences, USA), CD56 (NCAM 16.2; BD Biosciences, USA), CD123 (7G3; BD Biosciences, USA), CD127/IL-7α (A019D5; Biolegend, USA), CD197/CCR7 (3D12; BD Biosciences, USA), CD278/ICOS (DX29; BD Biosciences, USA), CD279/PD-1 (MIH4; BD Biosciences, USA) and HLA-DR (G46-6; BD Biosciences, USA), CD3 (17A2; Biolegend, USA), CD4 (V4; BD Biosciences, USA), CD8a (53-6.7; Biolegend, USA), CD11b (M1/70; Biolegend, USA), CD11c (HL3; BD Biosciences, USA), CD25 (PC61; Biolegend, USA), CD19 (SJ25C1; Biolegend, USA), CD24 (M1/69; Biolegend, USA), CD44 (IM7; BD Biosciences, USA), CD49b (HMα2; Biolegend, USA), CD62L (MEL-14; BD Biosciences, USA), CD69 (H1.2F3; Biolegend, USA), CD127 (A7R34; Biolegend, USA), CD206 (068C2; Biolegend, USA), CD278 (DX29; BD Biosciences, USA), CD335 (29A1.4; Biolegend, USA), FOXP3 (MF23; BD Biosciences, USA), Ly6C (HK1.4; Biolegend, USA), Ly6G (1A8; BD Biosciences, USA), NK 1.1 (PK136; BD Biosciences, USA), and NKG2D (CX5; Biolegend, USA) in 100 μl of FACS buffer for 30 min at 4°C. For staining of intracellular proteins (including transcription factors, cytokines and chemokines), FACS buffer supplemented with 10 μg/ml of BFA was used. The cells were stained with LIVE/DEAD fixable blue dead cell stain kit (Life Technologies, USA), primary antibodies and then fixed and permeabilized using the Fixation/Permeabilization staining kit (BD Cytofix/Cytoperm™; BD Biosciences, USA) as per manufacturer’s protocol. Staining was performed for 30 min at room temperature in the dark with the following antibodies, FOXP3 (206D; Biolegend, USA), IFN-*γ* (4S.B3; Biolegend, USA) and IL-10 (JES3-9D7; Biolegend, USA). Absolute count of cells in peripheral blood was determined using CountBright™ Absolute Counting Beads (ThermoFisher Scientific, USA). Data was acquired using a LSR II flow cytometer (BD Biosciences, USA) with FACSDiva software and analyzed using the Flowjo software version 10 (Treestar, USA).

### Cytokine and Chemokine Protein Quantification

A premixed LEGENDplex™ Human Inflammation Panel (13-plex) (Biolegend, USA) was used to measure plasma cytokine and chemokine levels. The 13 cytokines and chemokines assayed simultaneously include IL-1β, IFN-α, IFN-*γ*, TNF-α, MCP-1 (CCL2), IL-6, IL-8 (CXCL8), IL-10, IL-12p70, IL-17A, IL-18, IL-23, and IL-33. Samples, reagents, and immunoassay procedures were prepared and performed according to the manufacturer’s instructions. Data was acquired using a LSR II flow cytometer (BD Biosciences, USA) with FACSDiva software, and analysis was performed using LEGENDplex™ Data Analysis software (Biolegend, USA) based on standard curves plotted through a five-parameter logistic curve setting. Mice treated with Proleukin^®^/IL-2 had levels of IL-1β, IFN-α, TNF-α and IL-6 below detection limit and were excluded from subsequent analysis. For mice treated with OKT3, levels of IL-1β and IFN-α were below detection limit and excluded from analysis.

### T Cell Activation and ELISA

Mice were administered with a single dose of OKT3 and sacrificed 5 days later. EasySep™ PE Positive Selection Kit (Stem Cell Technologies, Vancouver, BC), CD4 PE (OKT4, Biolegend, USA) and CD8 PE (OKT4, Biolegend, USA) antibodies were used to isolate CD4^+^ and CD8^+^ T cells from blood (PBMCs) as per manufacturer’s instructions. Activation of CD4^+^ and CD8^+^ T cells was carried out with human T Cell Activation/Expansion Kit (Miltenyi, Germany) as per manufacturer’s instructions. After T cell stimulation, supernatant in the culture was harvested and analyzed with IFN-*γ (*Biolegend, USA) and TNF-*α (*Biolegend, USA) ELISA kits.

### Quantitative RT-PCR

RNA isolation was performed with RNeasy Mini and Micro kits (Qiagen, USA). Reverse transcription was performed using the iScript cDNA Synthesis Kit (Bio-Rad, USA) according to manufacturer’s specifications. All values were normalized with *β*-actin as an endogenous control. Data were analyzed with the comparative CT method in which gene expression is calculated as 2^−ΔΔCT^, where Delta Ct = (Ct gene of interest − Ct β-actin internal control). The primer sequences used are listed in **Supplementary Table 1**.

### Histology

Mouse organs were collected, fixed with 10% formalin and embedded in paraffin for processing into sections. Formalin-fixed paraffin sections (5 μm) were dewaxed by melting for 30 min at 65°C, cleared in xylene twice for 5 min, and rehydrated in water–ethanol solutions containing decreasing percentages of ethanol. To determine tissue morphology, sections were stained with hematoxylin–eosin (Gill 2 Hematoxylin and Eosin Y alcoholic; Thermo Sandon, UK) following standard procedures. For Immunohistochemistry, mouse organs were subjected to heat-mediated antigen retrieval with sodium citrate (pH6), incubated with anti-human CD45 (Abcam, UK) and stained with SuperPicture 3rd Gen IHC Detection Kit (Life Technologies, USA) according to the manufacturer’s instructions. Sections were imaged and analyzed under an Olympus BX-61 microscope (Olympus, Japan).

### Statistical Analysis

Statistical analysis was performed using GraphPad Prism 5.0 software (GraphPad Software Inc., USA). The correlation strength between the variables was assessed using the Spearman’s rank correlation coefficient. Pairwise comparison was performed using two-tailed Mann–Whitney *U* test/two-way Analysis of variance (ANOVA), *p* value less than 0.05 is considered statistically significant.

## Results

### Proleukin^®^/IL-2 Triggers an Inflammatory Response in Humanized Mice

**Figure 1:**
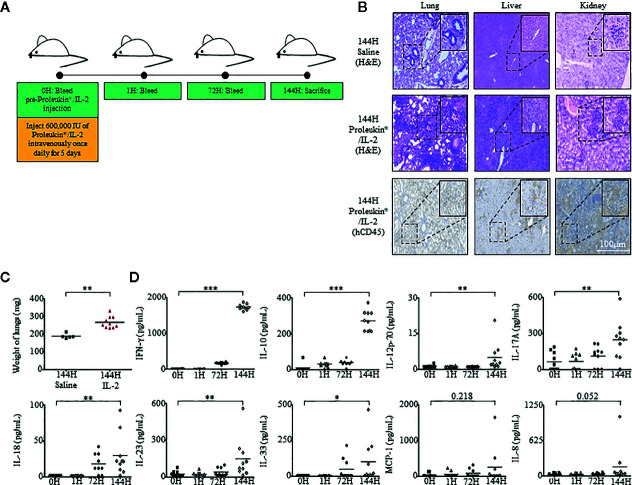
Humanized mice exhibit inflammatory responses when administered with Proleukin^®^/IL-2. **(A)** Adult humanized mice were bled at 0 h to obtain peripheral blood mononuclear cells (PBMCs) and plasma for baseline analysis (n = 15). Each mouse was injected with 600,000 IU of Proleukin^®^/IL-2 daily for 5 days (n = 10). Mice were bled at 1 h after Proleukin^®^/IL-2 administration, at 72 h before day 3 administration of Proleukin^®^/IL-2 and sacrificed at 144 h to collect organs, PBMCs and plasma for analysis. **(B)** Histological analysis of organs from both saline treated (n = 5) and Proleukin^®^/IL-2 treated (n = 10) humanized mice. Paraffin slides from indicated organs were processed and stained with H&E or anti-human CD45 antibody. Representative images are shown. Scale bar applies to all panels. **(C)** Weight of lungs from saline (n = 5) and Proleukin^®^/IL-2- (n = 10) treated humanized mice. Each symbol represents one mouse and the horizontal line indicates the mean value. **(D)** Human-specific cytokine and chemokine release of IFN-*γ*, IL-10, IL-12p70, IL-17A, IL-18, IL-23, IL-33, MCP-1, and IL-8 were measured in the plasma of humanized mice administered with saline (n = 5) or Proleukin^®^/IL-2 (n = 10). Each symbol represents one mouse and the horizontal line indicates the mean value. Two-tailed Mann–Whitney *U* test; (*p < 0.05, **p < 0.01, ***p < 0.001). Data are from a single experiment; one independent experiment was performed.

To assess *in vivo* immunotoxic effects of Proleukin^®^/IL-2, humanized mice were administered with Proleukin^®^/IL-2 (600,000 IU/mouse) once daily for 5 days (40, 41). Peripheral blood mononuclear cells (PBMCs) were collected from the mice at 0 hours (h), 1 h after first administration and 72 h. At 144 h (endpoint), the mice were sacrifice and their PBMCs and organs were harvested for analysis (**Figure 1A**). Histological analysis showed that the lungs, liver, and kidneys of Proleukin^®^/IL-2-treated humanized mice developed massive immune cell infiltrations (**Figure 1B**).

A life-threatening side effect of prescribing Proleukin^®^/IL-2 to patients is the development of pulmonary edema (23, 42–44). This condition is commonly attributed to pulmonary capillary permeability, which causes fluid build-up and an increase in hydrostatic pressure within a patient’s lung, in turn affecting air exchange leading to eventual death. To assess if humanized mice treated with Proleukin^®^/IL-2 experienced similar conditions to humans, we weighed the lungs at endpoint and observed that the lung weight of treated humanized mice were significantly heavier than that of saline-treated control mice (**Figure 1C**).

Administration of Proleukin^®^/IL-2 to patients usually results in “cytokine storm”, which is the release of cytokines at high levels, leading to many clinical symptoms (22, 45). To identify if humanized mice would respond by producing cytokine storms as akin to human subjects, we evaluated human cytokine and chemokine levels within the plasma of these mice at 0 h, 1 h after the first administration, 72 h, and at 144 h after the last administration of Proleukin^®^/IL-2. Similar to the clinical scenario (16), Proleukin^®^/IL-2 treatment in humanized mice induced an elevation of major chemokines and pro-inflammatory cytokines such as, IFN-*γ*, IL-12p70, IL-17A, IL-18, IL-23, IL-33, as well as an immunosuppressive cytokine, IL-10 at 144 h post-Proleukin^®^/IL-2 treatment but not at earlier time points (**Figure 1D**).

### Multiple Immune Cell Subsets Expand Drastically During Proleukin^®^/IL-2 Treatment

We performed a comprehensive multiparameter flow cytometry evaluation on PBMCs, spleen, and lymph nodes (**Supplementary Figure 1**) of humanized mice, at 0 and at 144 h (endpoint) after Proleukin^®^/IL-2, utilizing markers that covered both lymphoid (**Supplementary Figure 2**) and myeloid **(** **Supplementary Figures 3A–C**) cell subsets.

In clinical settings, ICOS^+^ regulatory T cells (CD4^+^CD25^+^FOXP3^+^ICOS^+^ Tregs) have been identified as a potential predicative biomarker to determine if a patient would be responsive to Proleukin^®^/IL-2 treatment (16). It has been reported that patients who produce high levels of ICOS^+^ Tregs during Proleukin^®^/IL-2 administration have an increased chance of responding to treatment (16). As shown in humanized mice, immunosuppressive cytokine IL-10 was produced in addition to chemokines and pro-inflammatory cytokines. Therefore, the frequency and phenotypes of human ICOS^+^ Tregs in Proleukin^®^/IL-2-treated humanized mice were analyzed.

**Figure 2:**
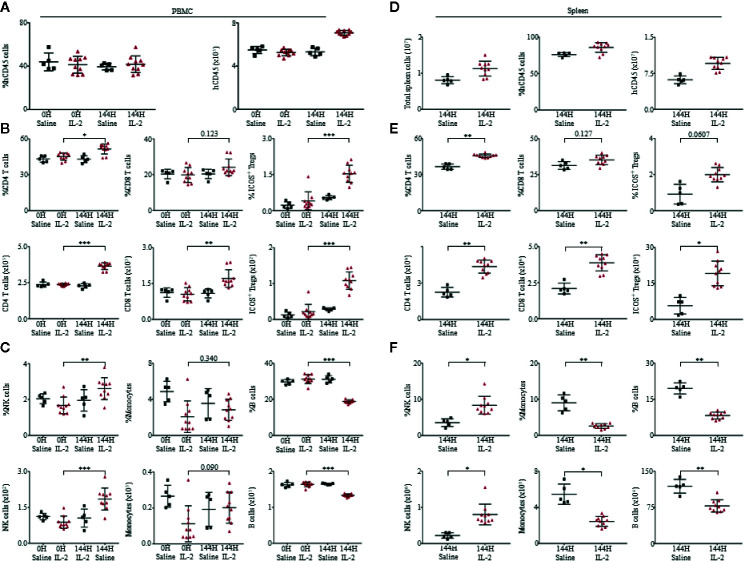
Proleukin^®^/IL-2 triggers expansion of human immune cell subsets. **(A)** Mean frequency of PBMCs and absolute count over human CD45^+^ cells ± SEM. Immunophenotypic analysis of **(B)** CD4^+^ T cells, CD8^+^ T cells, ICOS^+^ Treg, **(C)** NK cells, monocytes and B cell subsets within peripheral blood mononuclear cells (PBMCs) of saline (n = 5) and Proleukin^®^/IL-2 recipient mice (n = 10). **(D)** Mean frequency and absolute cell count of splenocytes are based on total human CD45^+^ cells ± SEM. Immune profile analysis of **(E)** CD4^+^ T cells, CD8^+^ T cells, ICOS^+^ Treg, **(F)** NK cells, monocytes and B cell subsets in splenocytes of saline (n = 5) and Proleukin^®^/IL-2 recipient mice (n = 10). Two-tailed Mann–Whitney *U* test; (*p < 0.05, **p < 0.01, ***p < 0.001). Data are from a single experiment; one independent experiment was performed.

Consistent to cytokine and chemokine levels in the plasma, there were minimal changes in the levels of immune cell subsets in PBMCs from 0 to 72 h, with most significant changes observed at 144 h. Spearman’s rank correlation coefficient revealed significantly positive correlation between the levels of CD45^+^ cells to the release of cytokines such as IFN-*γ* (*r* = 0.940*, p* < 0.001) and IL-10 (*r* = 0.903*, p* > 0.001) (**Supplementary Figure 3D**). Among the human immune cell subsets analyzed, CD4^+^ T cells, CD8^+^ T cells and ICOS^+^ Tregs expanded robustly, in terms of percentage relative to total live human leukocytes and absolute cell counts within PBMCs (**Figures 2A, B**) and spleen (**Figures 2D, E**) of humanized mice treated with Proleukin^®^/IL-2. There was also a significant increase in CD56^+^ natural killer (NK) cells within both PBMCs (**Figure 2C**) and spleen (**Figure 2F**) of Proleukin^®^/IL-2-treated humanized mice. Similar to clinical settings CD14^+^ monocytes and CD19^+^ B cells decreased in both PBMCs (**Figure 2C**) and splenocytes (**Figure 2F**) post-Proleukin^®^/IL-2 therapy (16).

To compare our findings in humanized mice to wild type mice, we administered C57BL/6 mice with the same dose of Proleukin^®^/IL-2 and observed that there was an increase in percentage of CD4^+^ T cells, CD8^+^ T cells, ICOS^+^ Tregs, activation of CD4^+^ and CD8^+^ T cells (by CD69^+^ upregulation) in PBMCs (**Supplementary Figure 4A**) and spleen (**Supplementary Figure 4B**) of these mice. While absolute cell counts of CD4^+^ T cells, CD8^+^ T cells, ICOS^+^ Tregs, monocytes, NK cells, B cells, and activation of CD4^+^ and CD8^+^ T cells (by CD69^+^ upregulation) increased in PBMCs (**Supplementary Figure 4A**), there was an increase in absolute cell counts of CD4^+^ T cells, CD8^+^ T cells, ICOS^+^ Tregs, monocytes, NK cells, B cells in the spleen (**Supplementary Figure 4B**). Even though C57BL/6 could also respond to Proleukin^®^/IL-2 with immune cell expansion and activation (by upregulation of CD69), it lacked human-specific responses for example, the expression of MHC-II on activated T cells. This study strongly supports that humanized mice can more accurately recapitulate clinical responses and should be used to reduce the total number of animals utilized for research and clinical trials.

### Proleukin^®^/IL-2 Activates T Cells Toward Terminally Differentiated Phenotype

**Figure 3:**
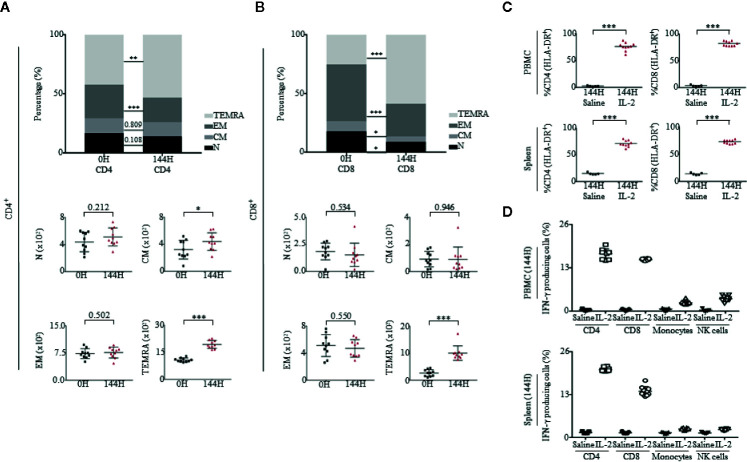
Proleukin^®^/IL-2 activates T cells toward memory phenotypes. **(A, B)** Immunophenotypic analysis of both CD4 and CD8 subsets of Naïve (N; CD45RA^+^CCR7^+^), central memory (CM; CD45RA^–^CCR7^+^), effector memory (EM; CD45RA^−^CCR7^−^) and effector memory RA (TEMRA; CD45RA^+^CCR7^−^) in PBMCs at 0 and 144 h of mice administered with Proleukin^®^/IL-2 (n = 10). Mean frequency of (**A**, top panel) CD4^+^ T cells and (**A**, bottom panel) absolute count over total human CD45^+^ cells ± SEM. Mean frequency of (**B**, top panel) CD8^+^ T cells and (**B**, bottom panel) absolute count of human CD45^+^ cells ± SEM. **(C)** Percentage of CD4^+^HLA-DR^+^ and CD8^+^HLA-DR^+^ cells in the (**C**, top panel) PBMCs and (**C**, bottom panel) spleen of saline (n = 5) and Proleukin^®^/IL-2-treated mice (n = 10) at 144 h. **(D)** Percentage of IFN-*γ* was measured within T cells in (**D**, top panel) PBMCs and (**D**, bottom panel) spleen at 144 h of saline (n = 5) and Proleukin^®^/IL-2-treated mice (n = 10), injected with BFA. Two-way ANOVA/Two-tailed Mann–Whitney *U* test; (*p < 0.05, **p < 0.01, ***p < 0.001). Data are from a single experiment; one independent experiment was performed.

As T cells expanded in response to Proleukin^®^/IL-2, we further investigated the immunophenotype of both CD4^+^ and CD8^+^ T cells. After Proleukin^®^/IL-2 administration, both CD4^+^ (**Figure 3A**) and CD8^+^ T cells (**Figure 3B**) lost characteristics of Naïve (N; CD45RA^+^CCR7^+^) and effector memory (EM; CD45RA^−^CCR7^−^) phenotypes. Instead, T cells with effector memory RA (TEMRA; CD45RA^+^CCR7^−^) increased. We examined the activation status of CD4^+^ and CD8^+^ T cells by analyzing the expression of HLA-DR, a molecule upregulated upon human T cell activation not usually observed in mice. Proleukin^®^/IL-2 treatment activated CD4^+^ and CD8^+^ T cells to upregulate HLA-DR expression in both PBMCs and spleen (**Figure 3C**). To detect intracellular cytokines, mice were injected with Brefeldin A (BFA). Upon activation, both CD4^+^ and CD8^+^ T cells had a greater number of IFN-*γ* producing cells as compared to monocytes and NK cells (**Figure 3D**). Together with absolute cell numbers, the cytokine production data confirmed that T cells were the main effector cells for the immunotoxicity induced by Proleukin^®^/IL-2.

### ICOS^+^ Tregs Have Phenotypic Characteristics of Tregs

In published clinical studies, FOXP3 expression has been associated in T cells with a regulatory or suppressive phenotype (46, 47). In some situations, a transient expression of FOXP3 at low levels can occur when effector, non-Treg CD4^+^ cells are activated (48, 49). Prior to Proleukin^®^/IL-2 treatment, Tregs were ICOS^−^;, however, after Proleukin^®^/IL-2 therapy, ICOS^+^ Tregs increased drastically. Therefore, we investigated if these cells had immunosuppressive and regulatory phenotypic characteristics of Tregs seen in patients and were not merely non-Treg CD4^+^FOXP3^+^ T cells with an ICOS activation marker (16).

**Figure 4:**
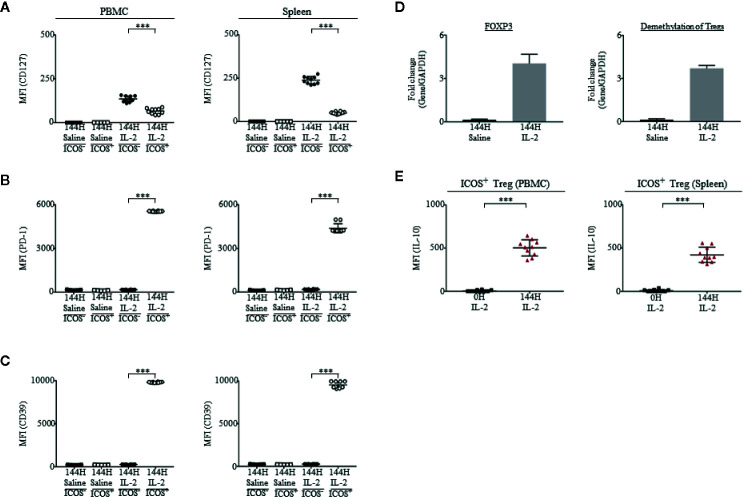
Tregs from mice administered with Proleukin^®^/IL-2 display highly immunosuppressive phenotypes. **(A–C)** In saline treated (n = 5) mice, Tregs were ICOS^−^. ICOS was upregulated on Tregs upon Proleukin^®^/IL-2 treatment (n = 10) (**Supplementary Figure 2B**). Mean fluorescence intensity (MFI) in PBMCs and splenocytes, illustrating the expression of **(A)** CD127, **(B)** PD-1 and **(C)** CD39 on ICOS^+^ and ICOS^−^ Tregs in saline treated mice and ICOS^+^ and ICOS^−^ Tregs in Proleukin^®^/IL-2-treated mice at 144 h was shown. **(D)** Transcript expression of FOXP3 and demethylation of Tregs 144 h post saline (n = 5) and IL-2 treatment (n = 10). **(E)** Mean fluorescence intensity (MFI) in PBMCs and splenocytes, illustrating the production of IL-10 by ICOS^+^ Treg after IL-2 administration. Two-tailed Mann–Whitney *U* test; (*p < 0.05, **p < 0.01, ***p < 0.001). Data are from a single experiment; one independent experiment was performed.

First, in accordance with previous studies (50–54), we observed the staining of CD25 and FOXP3 on ICOS^+^ and ICOS^−^ cells. Cells which were ICOS^+^ had a higher intensity staining of CD25 and FOXP3 than ICOS^−^ cells (**Supplementary Figure 2**). Second, ICOS^+^ Tregs expressed low levels of CD127/IL-7*α* in both PBMCs and spleen of humanized mice (**Figure 4A**). Third, we observed the expression of programmed cell death protein 1 (PD-1) and found that ICOS^+^ Tregs had a higher level of PD-1 than ICOS^−^ Tregs in both PBMCs and spleen of humanized mice (**Figure 4B**). Fourth, studies have linked highly suppressive Tregs to CD39 ectonucleotidase expression (55–57). In our experiment, we observed that ICOS^+^ Tregs were CD39^+^ while non-Tregs were CD39^−^, which was similar in both PBMCs and spleen of humanized mice (**Figure 4C**). Fifth, at 144 h post Proleukin^®^/IL-2 treatment, mice had heightened expression of FOXP3 and demethylation of Treg-specific-demethylated region (TSDR), suggesting activation of Tregs (**Figure 4D**). Lastly, it has been demonstrated that suppressive ICOS^+^ Tregs are able to secrete high levels of IL-10 and low levels of IFN-*γ* in response to stimulation. In agreement with published findings (16, 58), the intracellular cytokine staining revealed that ICOS^+^ cells produced high levels of IL-10 (**Figure 4E**) but did not produce IFN-*γ* at 144 h post-Proleukin^®^/IL-2 stimulation in both PBMCs (**Supplementary Figure 2B**) and spleen (**Supplementary Figure 2C**) of humanized mice. These results indicate that expanded ICOS^+^ Tregs in humanized mice treated with Proleukin^®^/IL-2 treatment display phenotypic attributes of Tregs similar to clinical scenarios (16).

### OKT3 Induces Immunotoxic Effects in Humanized Mice

**Figure 5:**
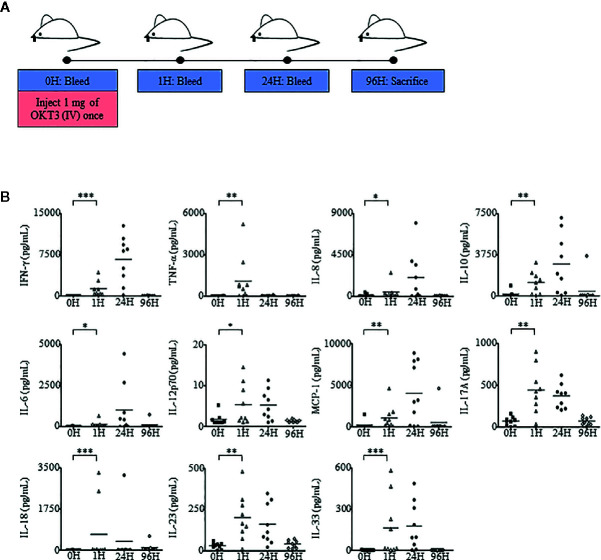
Humanized mice administered with OKT3 exhibit heightened cytokine and chemokine production. **(A)** Adult humanized mice were bled at 0 h to obtain PBMCs and plasma for baseline analysis (n = 9). Each mouse was injected with 1 mg of OKT3 only once, bled at 1 h after OKT3 administration, 24 h and sacrificed at 96 h (endpoint). Organs, PBMCs, and plasma were collected, processed, and analyzed. **(B)** Human-specific cytokine and chemokine release of IFN-*γ*, TNF-α, IL-6, IL-8, IL-10, IL-12p70, MCP-1, IL-17A, IL-18, IL-23, and IL-33 were measured in the plasma of humanized mice administered with OKT3 (n = 9). Each symbol represents one mouse and the horizontal line indicates the mean value. Two-tailed Mann–Whitney *U* test; (*p < 0.05, **p < 0.01, ***p < 0.001). Data are from a single experiment; one independent experiment was performed.

Besides cytokine treatments such as Proleukin^®^/IL-2, antibody therapy can also induce Immunotoxicity (31, 59). To determine whether humanized mice were able to recapitulate antibody-mediated immunotoxicity in humans, we evaluated a T cell receptor (TCR) triggering antibody, OKT3 (anti-CD3) in humanized mice. Humanized mice were analyzed at 0 h to obtain baseline levels of cytokines, chemokines, and immune cell subsets. As per clinical practice, in contrast to Proleukin^®^/IL-2 treatment which usually requires a longer duration of treatment to observe side effects, OKT3-mediated immunotoxicity occurs within a shorter time frame (60, 61). Mice were analyzed at 1 h post-administration of OKT3, 24 h, and sacrificed 96 h later (**Figure 5A**). Human chemokine and cytokine analysis in plasma showed that human chemokines and pro-inflammatory cytokines, such as IFN-*γ*, TNF-α, IL-6, IL-8, MCP-1, IL-17A, IL-18, IL-23, IL-33 and an immunosuppressive cytokine IL-10, started to elevate at 1 h post-OKT3 treatment (**Figure 5B**) with some cytokines and chemokines, IFN-*γ*, IL-8, IL-6, MCP-1 and IL-10 reaching its peak at 24 h. Most of the cytokines and chemokines returned to baseline levels by 96 h post-OKT3 treatment. Spearman’s rank correlation coefficient revealed that there was a significant positive correlation between CD45^+^ cell levels and cytokines including IFN-*γ* (*r* = 0.950*, p* < 0.001) and IL-10 (*r* = 0.933*, p* < 0.001) production (**Supplementary Figure 5C**). These results demonstrated that similar to clinical data, cytokine storm induced by immunotoxic antibody can be reproduced in humanized mice.

**Figure 6:**
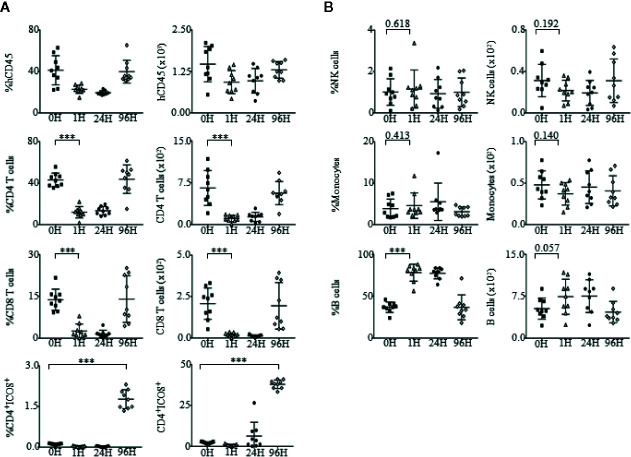
Changes in immune cell subsets upon administration of OKT3. **(A)** Immunophenotypic analysis of CD4^+^ T cells, CD8^+^ T cells and CD4^+^ICOS^+^ T cell subsets within the PBMCs of OKT3 recipient mice (n = 9). (**A**, left panel) Mean frequency and (**A**, right panel) absolute count of CD4^+^ T cells, CD8^+^ T cells and CD4^+^ICOS^+^ T cells of total human CD45^+^ cells ± SEM. **(B)** Immune profile analysis of NK cells, monocytes and B cell subsets within the PBMCs of mice administered with OKT3. (**B**, left panel) Mean frequency and (**B**, right panel) absolute count of NK cells, monocytes, and B cells over total human CD45^+^ cells ± SEM. Two-tailed Mann–Whitney *U* test; (*p < 0.05, **p < 0.01, ***p < 0.001). Data are from a single experiment; one independent experiment was performed.

We further examined the effects of OKT3 stimulation on the regulation of immune cell subsets such as, CD4^+^ T cells, CD8^+^ T cells, NK cells, monocytes, and B cells (**Supplementary Figure 5**) within PBMCs (**Figures 6A, B** **)** and splenocytes (**Supplementary Figure 6**) of humanized mice. In addition, as it has been reported that upregulation of ICOS is greatly dependent on TCR activation (62), the involvement of CD4^+^ICOS^+^ T cells was also analyzed. At 24 h post-OKT3 treatment, humanized mice had drastically reduced frequencies and absolute numbers of CD4^+^ and CD8^+^ T cells due to the depletion effects of OKT3 antibody to CD3 T cells. However, the frequencies and number of CD4^+^ and CD8^+^ T cells recovered to baseline levels by 96H post-treatment, and there was an increase in CD4^+^ICOS^+^ T cells at 24 to 96 h post-OKT3 administration (**Figure 6A**). Treatment of humanized mice with OKT3 did not result in changes to the actual cell counts of NK cells and monocytes (**Figure 6B**).

**Figure 7:**
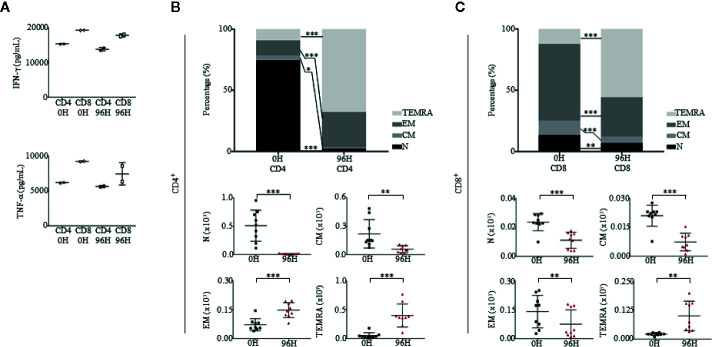
Humanized mice treated with OKT3 have functional T cells and express altered CD4 and CD8 differentiation profiles. **(A)** T cells were stimulated with human T Cell Activation/Expansion Kit and levels of human IFN-*γ* and TNF-α were measured by ELISA (n = 2). **(B, C)** Immunophenotypic analysis of both CD4 and CD8 subsets of Naïve (N; CD45RA^+^CCR7^+^), central memory (CM; CD45RA^-^CCR7^+^), effector memory (EM; CD45RA^−^CCR7^−^) and effector memory RA (TEMRA; CD45RA^+^CCR7^−^) in PBMCs at 0H and 96H of mice administered with OKT3 (n = 9). (**B**, top panel) Mean frequency of CD4^+^ T cells and (**B**, bottom panel) absolute count based on total human CD45^+^ cells ± SEM. (**C**, top panel) Mean frequency of CD8^+^ T cells and (**C**, bottom panel) absolute count over total human CD45^+^ cells ± SEM. Two-way ANOVA/Two-tailed Mann–Whitney *U* test; (*p < 0.05, **p < 0.01, ***p < 0.001). Data are from a single experiment; one independent experiment was performed.

To evaluate the functionality of T cells in pre-treated (0 h) and post-treated OKT3 (96 h) mice, we isolated CD4^+^ and CD8^+^ T cells and stimulated them with a T cell activation/expansion kit. Post-activation, the supernatant of CD4^+^ and CD8^+^ stimulated T cells was harvested and tested for the presence of IFN-*γ* and TNF-α *via* ELISA. Even though levels of IFN-*γ* and TNF-α were slightly lower in 96 h CD4^+^ and CD8^+^ T cells as compared to 0 h, the production of cytokines suggest that T cells that returned to the blood after OKT3 treatment were functional (**Figure 7A**). Although T cell numbers recovered 96 h post-OKT3 treatment, the composition of T cell subsets had changed significantly. Both CD4^+^ T cells (**Figure 7B**) and CD8^+^ T cells (**Figure 7C**) developed distinctive terminally differentiated phenotypes where percentages and absolute numbers of N, CM, and EM cells decreased while TEMRA cells were significantly elevated. These results from humanized mice correlate to published studies on patient responses (16, 27, 28, 63).

## Discussion

A pivotal reason for inferior success rates of biologics evaluated during clinical development may be due to conventional application of *in vitro* tests and non-human animal models used to evaluate biologics specific for human targets during pre-clinical development (9, 10). Currently, the most suitable species for preclinical safety assessment of biologics are non-human primates (NHPs); however, the use of these animals raise critical ethical and economic issues including heighten level of sentience, scarcity, and high costs (35). In addition, analysis of NHPs demonstrated differences in biological systems particularly that of the immune system, resulting in inconsistent correlation of possible immunotoxic side effects when tested with biologics designed with human-specific targets (5, 7, 64). For example, the use of cynomolgus and rhesus monkeys to screen for side effects of TGN1412 showed extremely different safety and efficacy profiles as compared to humans due to their dissimilarities in CD28 sequence and molecule (38, 64–66). Shortcomings in these assessments, suggest that the selection of a cost-effective, validated, predictive animal model, containing human-specific cells will add substantial value in identifying *in vivo* human-specific clinical toxicity, particularly for biologics with novel mechanisms of action, therefore allowing accurate safety assessment, determination of exact dose and timing of administration to patients (5, 8, 67).

In published studies, research teams constructed different humanized mice by using a myriad of immunodeficient mouse strains, reconstituted with human PBMCs, cord blood or fetal liver. Some of these humanized mice have been applied as a platform to test plasmids, antibodies and biologics (68–75). For example, Li et al. tested IL-2 plasmid and CD3 antibody in human immune system (HIS) mice [generated in BALB/cR-ag2^−/−^ Il2r-*γ* ^−/−^ SIRPA^NOD^ (BRGS) hosts] reconstituted with fetal liver CD34^+^ cells (75). Different from published work, we utilized one of the most well-studied and accessible immunodeficient mouse strain, NSG. To prevent unwanted side effects that have been reported with the use of PBMCs, we reconstituted mice with human fetal liver stem cells and recapitulated clinical scenarios closely by using the exact clinical drug, dose and administration method to evaluate the effects of Proleukin^®^/IL-2 in humanized mice (40).

In this study, we have shown that mice reconstituted with a human immune system, more accurately recapitulated human immune responses to immunotoxic biologics. Humanized mice treated with Proleukin^®^/IL-2 for 5 consecutive days at a dose of 600,000 IU demonstrated an increase in total lung weight, immune cell infiltration into organs, elevated levels of chemokines and pro-inflammatory cytokines, IFN-*γ*, IL-12p70, IL-17A, IL-18, IL-23 and IL-33 and an immunosuppressive cytokine, IL-10 (15, 76). Additionally, percentages and absolute numbers of CD4^+^ T cells, CD8^+^ T cells, ICOS^+^ Tregs and NK cells were upregulated, while levels of monocytes and B cells decreased (15, 21, 26, 76). To further validate this model, we administered an anti-CD3 antibody—OKT3—to humanized mice and observed that massive amounts of chemokines and pro-inflammatory cytokines, IFN-*γ*, TNF-α, IL-6, IL-8, MCP-1, IL-17A, IL-18, IL-23, and IL-33 were secreted predominantly at 1 h post-treatment (77, 78). At 96 h, levels of T cells gradually recovered; this phenomenon could be due to a homeostatic mechanism to maintain constant T cell numbers in the circulation known as lymphopenia-induced proliferation (LIP) (79). Interestingly, in addition to inflammatory responses, which were considered to be the main cause of immunotoxicity, immunosuppressive responses, such as the expansion of ICOS^+^ Treg cells and production of immunosuppressive cytokine, IL-10, occurred concurrently. These results suggest that immunotoxicity may be a competitive dynamic process between inflammation and immunosuppression (80). Besides the effort on reducing inflammation (44), development of strategies to enhance immunosuppression may provide new options to the treatments of an array of conditions and diseases (81, 82). Most importantly, our results are in concordance with that of published clinical studies (16), therefore enabling the use of humanized mice as a pre-clinical platform to determine human-specific biologic mechanisms of action.

Despite the advantages and significant findings from using humanized mice, limitations in this work include firstly, the measurement of lung total weight which could have been further optimized by analyzing lung wet weight so as to differentiate between intrapulmonary cellular infiltrates and lung edema (17). Secondly, TNF-α was not upregulated in Proleukin^®^/IL-2-treated humanized mice (18–20). Possible reasons for this observation could be that our current model relies on the transplantation of HSPCs which do not give rise to endothelial cells of human-origin. Endothelial cells together with IL-2 receptor-positive immune cells are essential for the release of vasoactive factors such as TNF-α (18–20). In addition, our model requires further optimization to improve levels of human cell engraftment and functionality of immune subsets such as myeloid cells, which are crucial for TNF-α production but are relatively low in the current model (83). In recent years, humanized mouse strains such as NSG-SGM3, HuNOG-EXL, and MISTRG mice have been created with improved human immune cell reconstitution (84). However, despite the increase of reconstitution in these mice, drawbacks include the need for matched donors to create thymus, liver, and hematopoietic stem cell engrafted mice (NSG-SGM3, HuNOG-EXL), human immune cell reconstitution that lasts not more than 5 months (NSG-SGM3), anemia and short lifespan after human cell engraftment (HuNOG-EXL, MISTRG), undetermined long-term effects of irradiation tolerance and incidences of lymphoma (NSG-SGM3, HuNOG-EXL, MISTRG) and impaired innate immune cells including macrophages and dendritic cells (NSG-SGM3, HuNOG-EXL, MISTRG) (85–88). To improve current versions of humanized mice, murine cells should be completely depleted to allow an increase in human cell reconstitution which can be supplemented by hydrodynamically boosting mice with high affinity cytokines (*e.g.* IL-1β, IL-2, IL-7, and GM-CSF) to induce immune cell differentiation, maturation and function (83, 89, 90). Overcoming obstacles and improving humanized mice would undoubtedly provide exciting opportunities for the scientific community to evaluate novel therapeutics including biologics that would greatly benefit society.

Outcomes for specific therapeutic developments are largely dependent on selected animal models which allow the validation of therapeutic mechanisms and prediction of clinical efficacy (5, 91). When properly designed and carried out, animal models can contribute essential information to our knowledge of biology and medicine, including the discovery and development of new therapies (1, 2). However, due to the evolutionary gap between species, existing animal models are unable to fully recapitulate human biological responses, particularly that of the immune system (5). Advantages of utilizing humanized mice are its ability to more accurately mimic and reproduce human-specific responses (92, 93) for example, allowing the test of biologics specific to human CD3, production of human cytokine, IL-8 and the expression of HLA-DR on activated T cells, as demonstrated in this study. Therefore, in addition to current *in vitro* and *in vivo* pre-clinical tests, we advocate the use of humanized mice as a key for successful and safe clinical translation, so as to develop, optimize, validate, and create treatments.

In summary, humanized mice are small, easy to manipulate, cost-effective and robust platform that recapitulates the immune responses of human subjects. Because of the similarities in cellular and molecular mechanisms between humanized mice and humans (13, 84, 94), it unquestionably provides valuable data on the clinical performance of treatments, allowing researchers to learn human immunological mechanisms or be alerted of potential adverse effects of therapeutics before testing them on human subjects, therefore enabling the translation of a plethora of treatments which will benefit patients.

## Data Availability Statement

All datasets presented in this study are included in the article/**Supplementary Material**.

## Ethics Statement

The studies involving human participants were reviewed and approved by SingHealth and National Health Care Group Research Ethics Committees Singapore. The patients/participants provided their written informed consent to participate in this study. The animal study was reviewed and approved by International Animal Care and Use Committee (IACUC), A*STAR.

## Author Contributions

KY designed and performed experiments, analyzed and interpreted data, and prepared the manuscript. ZH, ST, WT, ML, FL, and SH performed experiments. YF, KC, C-IW, MK, JKYC, and JC provided the research tools, were involved in discussions, and prepared the manuscript. QC conceived the study, supervised the project and prepared the manuscript. All authors contributed to the article and approved the submitted version.

## Funding

This work was supported by the following grants: National Medical Research Council Singapore, VICTORY programme (NMRC/OFLCG/003/2018) and CS-IRG (CIRG19may-0051), A*STAR IAF-PP IMPACT programme (H18/01/a0/022) and National Research Foundation Fellowship Singapore NRF-NRFF2017-03, Competitive Research Programme NRF2016-CRP001-103 and NRF2019-NRF-ISF003-3127 to QC.

## Conflict of Interest

The authors declare that the research was conducted in the absence of any commercial or financial relationships that could be construed as a potential conflict of interest.

## Supplementary Material

Supplementary Table 1 Primers for qPCR. Primer sequences for FOXP3-TSDR demethylation-specific, FOXP3-TSDR methylation-specific, FOXP3, and *β*-actin.

## Abbreviations

BFA, Brefeldin A; CM, Central memory; EM, Effector memory; IFN-*γ*, Interferon-γ; N, Naïve; NK, Natural Killer; PBMCs, Peripheral blood mononuclear cells; PD-1, Programmed cell death protein 1; SEM, Standard error of mean; TCR, T cell receptor; TEMRA, Effector memory RA; TNF-α, Tumor necrosis factor alpha.
