# Supplementary material for: Humanized Mouse as a Tool to Predict Immunotoxicity of Human Biologics
Source: Front Immunol. 2020 Oct 15;11:553362. doi: 10.3389/fimmu.2020.553362 (PMC7604536; doi:10.3389/fimmu.2020.553362)
Supplement: Supplementary file 1 [file DataSheet_1.pdf]

Yong K et al. Supplementary Figure 1

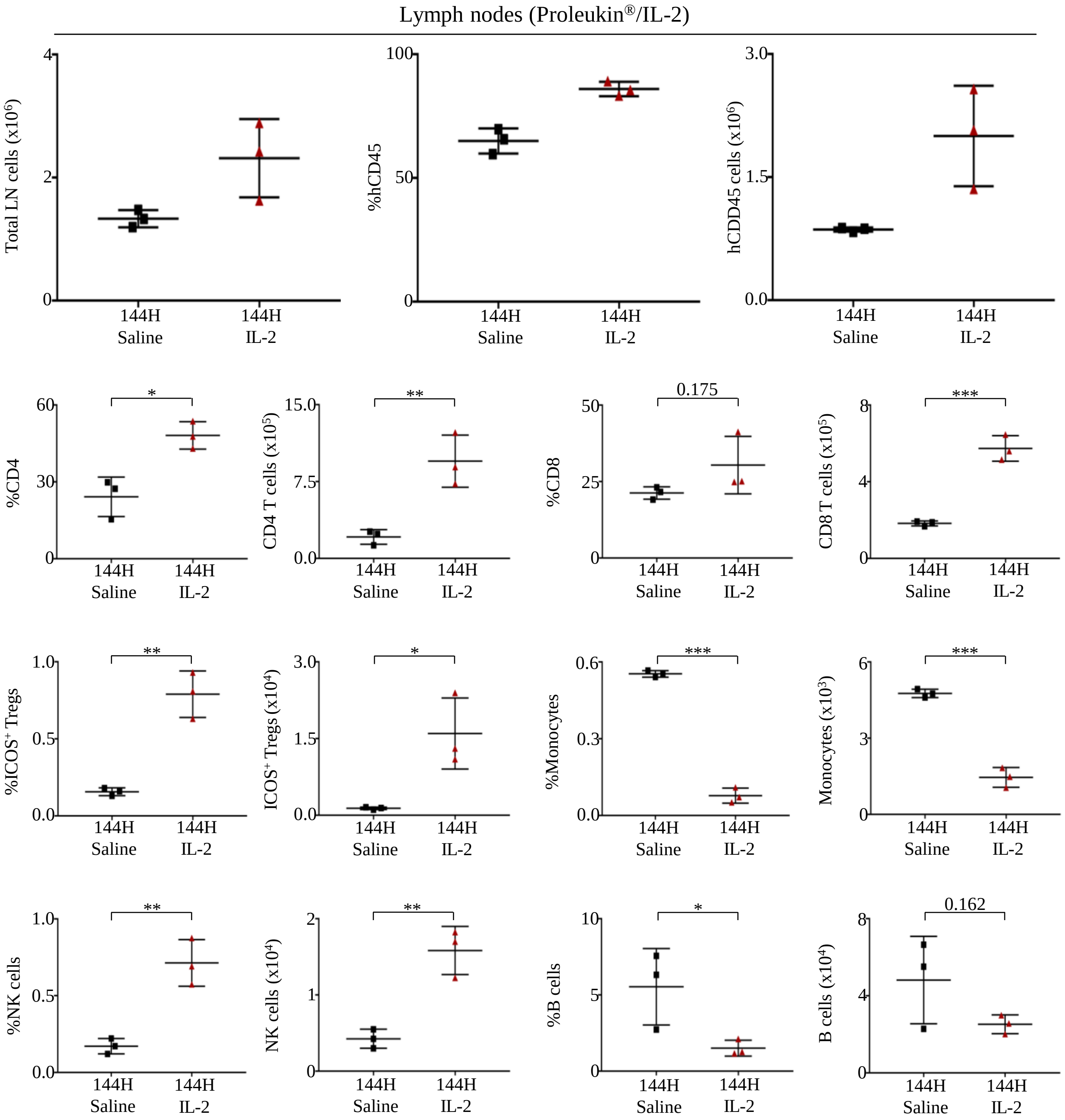

Supplementary Figure 1. Proleukin®/IL-2 triggers expansion of human immune cell subsets in lymph nodes. Comparison of $CD4^+$ T cells, $CD8^+$ T cells, $CD4^+ICOS^+$ T cell subsets, NK cells, monocytes and B cell subset profile within the lymph nodes of saline and Proleukin®/IL-2 recipient mice (n=3 per group) at 144H post-treatment. Average lymph node cell count, average absolute count and mean frequency of immune cells relative to total human $CD45^+$ cells ± SEM are presented. Two-tailed Mann Whitney *U* test; (*$p < 0.05$, **$p < 0.01$, ***$p < 0.001$). Data are from a single experiment; one independent experiment was performed.

Yong K et al. Supplementary Figure 2

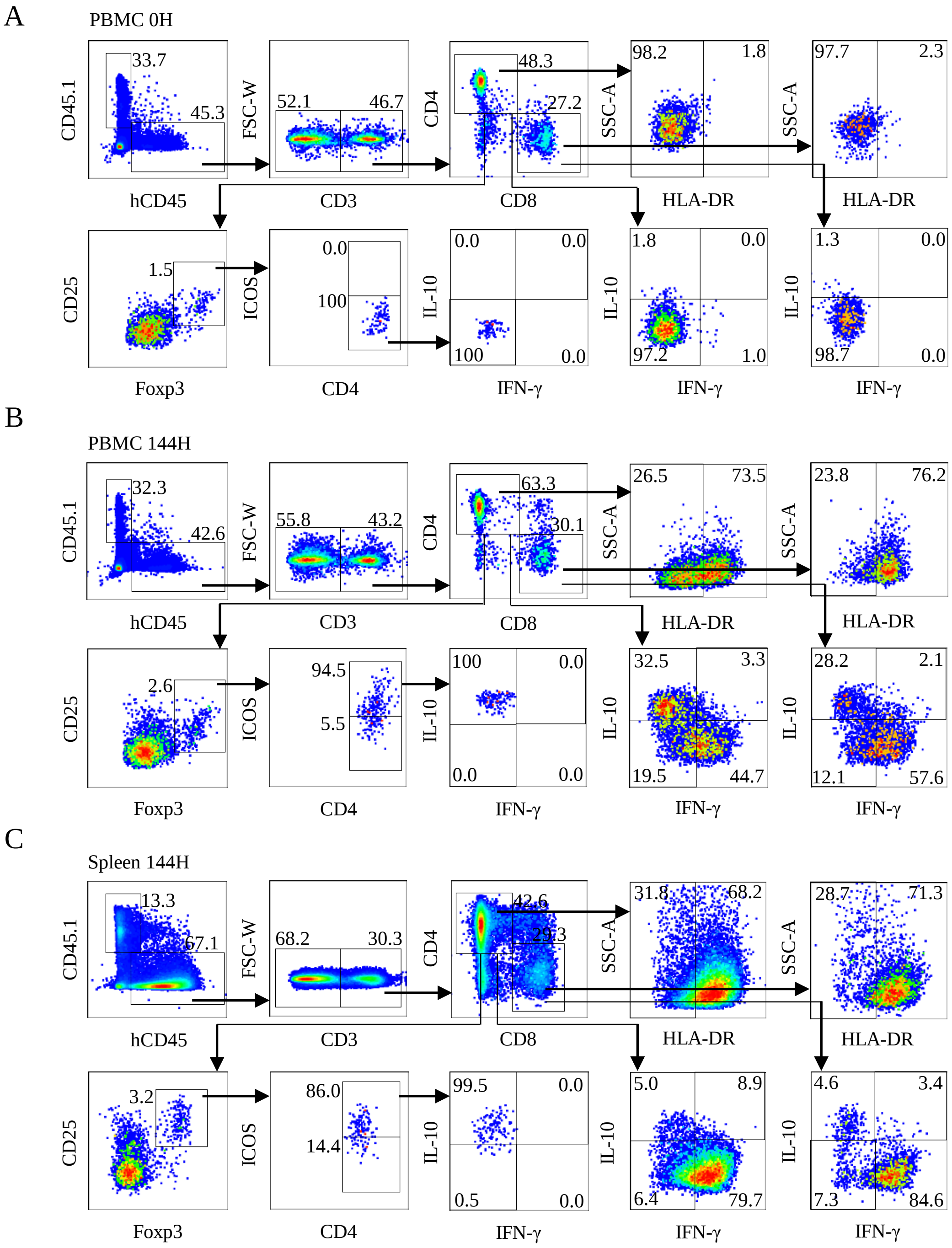

Supplementary Figure 2. Phenotype of T cells from Proleukin®/IL-2 treated humanized mice. (**A-C**) Immune profile of peripheral blood mononuclear cells (PBMCs) and splenocytes from humanized mice treated with Proleukin®/IL-2. Mononuclear cells isolated from humanized mice were immunolabeled with mouse CD45.1, human CD45, CD4, CD8, HLA-DR, CD25, FOXP3, ICOS, IL-10, and IFN-γ and analyzed using flow cytometry. Representative flow cytometry plots illustrating the expression of $CD4^+$, $CD8^+$, $CD4^+HLA\text{-}DR^+$, $CD8^+HLA\text{-}DR^+$, $CD4^+CD25^+FOXP3^+$, $CD4^+CD25^+FOXP3^+ICOS^+$, $CD4^+CD25^+FOXP3^+ICOS^-$, $IL\text{-}10^+$ and $IFN\text{-}\gamma^+$ for (**A**) PBMCs at 0H, (**B**) PBMCs at 144H and (**C**) splenocytes at 144H are shown. The numbers illustrated are the frequency of gated cells relative to parent population.

Yong K et al. Supplementary Figure 3

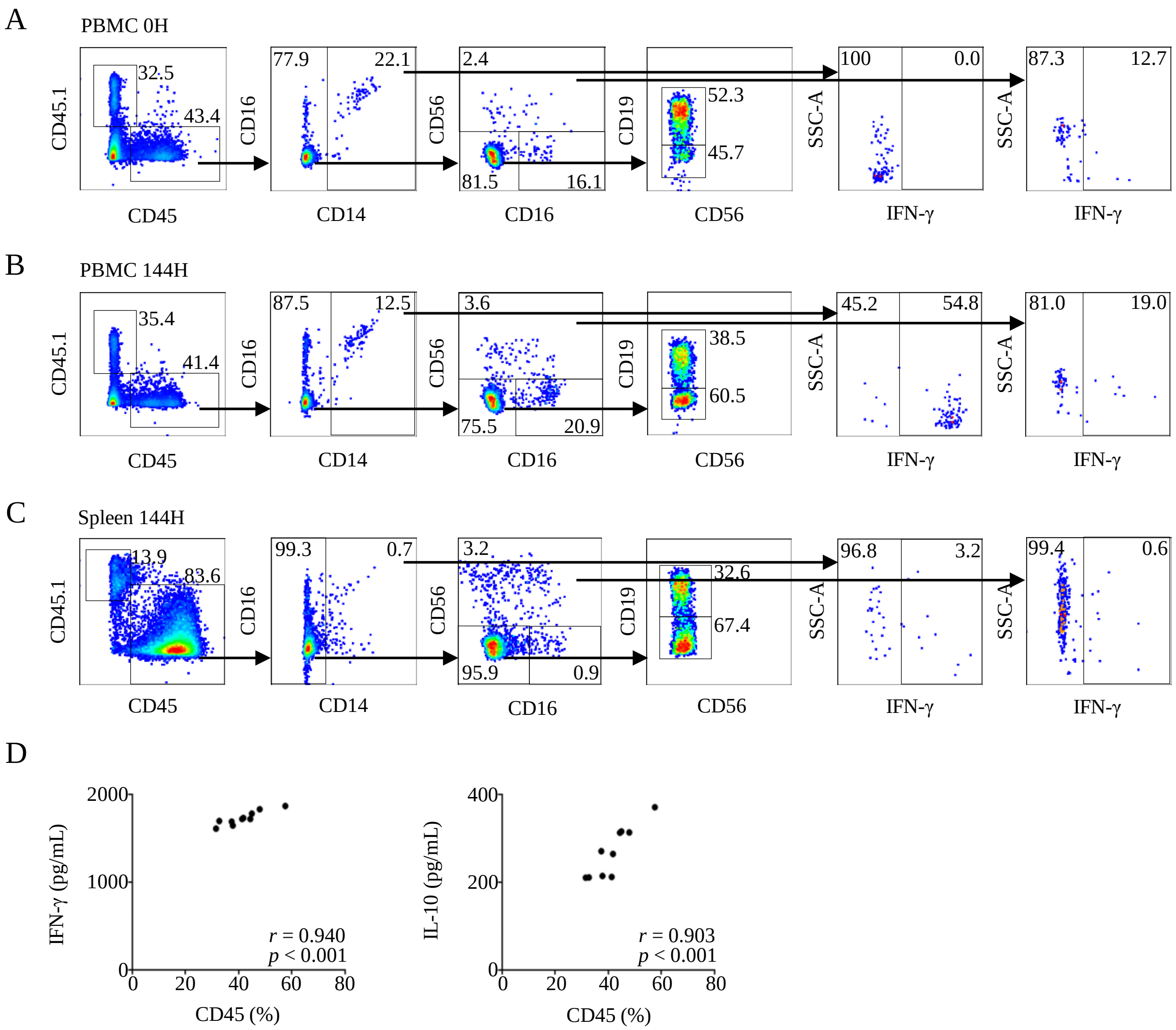

Supplementary Figure 3. Phenotype of non-T cells from Proleukin®/IL-2 treated humanized mice and correlation of cytokine production with human immune cell levels. (**A-C**) Immune profile of peripheral blood mononuclear cells (PBMCs) and splenocytes cells from humanized mice treated with Proleukin®/IL-2. Mononuclear cells isolated from humanized mice were immunolabeled with mouse CD45.1, human CD45, CD14, CD16, CD19, CD56, and IFN-γ and analyzed using flow cytometry. Representative flow cytometry plots illustrating the expression of monocytes, NK cells, B cells and IFN-γ subsets for (**A**) PBMCs at 0H, (**B**) PBMCs at 144H and (**C**) splenocytes at 144H are shown. The numbers illustrated are the frequency of the gated cells relative to the parent population. (**D**) Spearman's rank correlation coefficient analysis between the levels of human $CD45^+$ cells and IFN-γ and IL-10 at 144H post-IL-2 treatment (n=10).

Yong K et al. Supplementary Figure 4

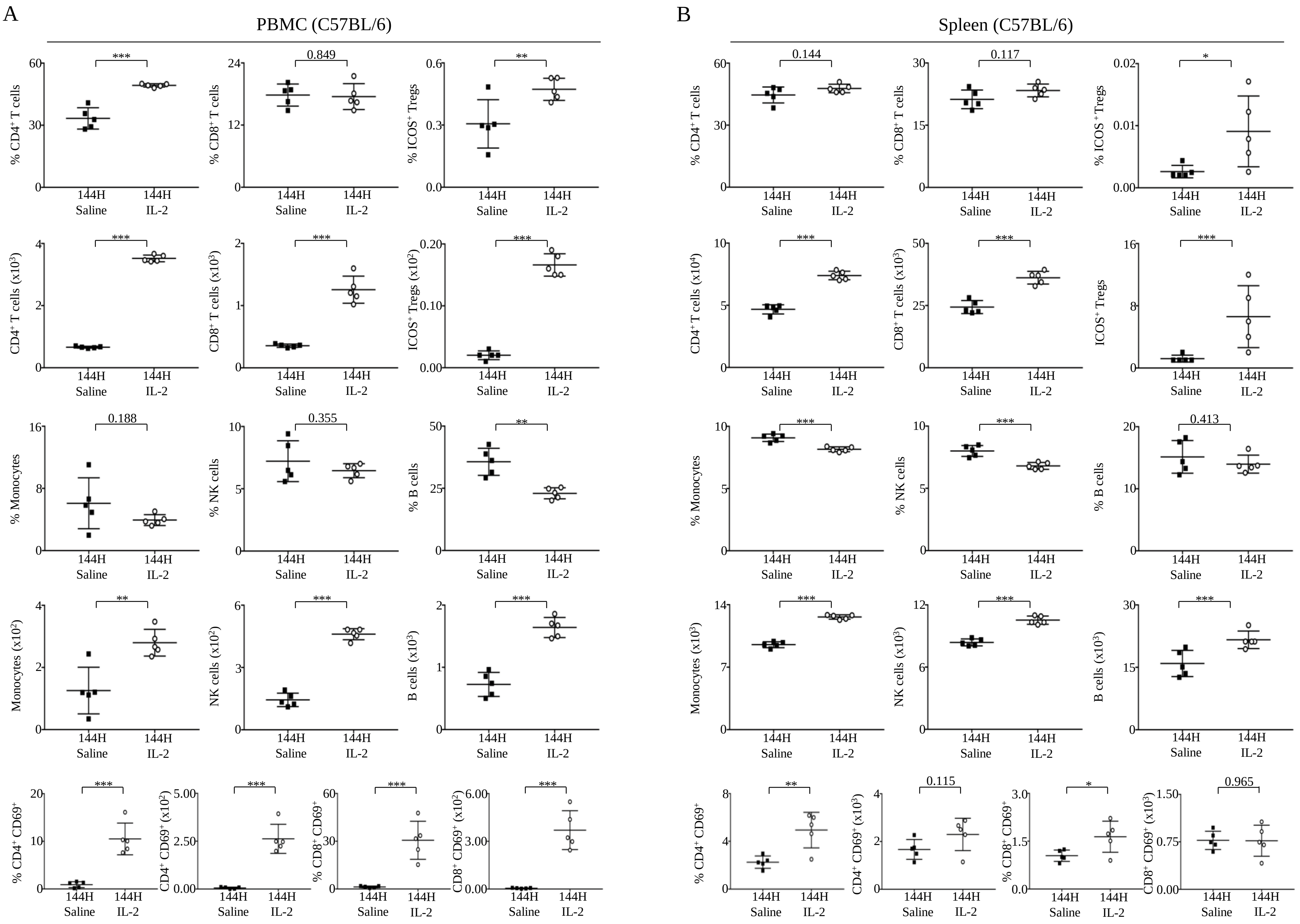

Supplementary Figure 4. Proleukin®/IL-2 triggers expansion of mouse immune cells in C57BL/6 model. (**A-B**) Comparison of mouse $CD4^+$ T cells, $CD8^+$ T cells, $CD4^+ICOS^+$ T cell subsets, NK cells, monocytes, B cell subsets, $CD4^+CD69^+$ and $CD8^+CD69^+$ profiles within the (**A**) blood and (**B**) spleen of saline and Proleukin®/IL-2 recipient mice (n=5 per group) at 144H post treatment. Average absolute count and mean frequency of immune cells relative to mouse CD45 ± SEM are shown. Two-tailed Mann Whitney *U* test; (*$p < 0.05$, **$p < 0.01$, ***$p < 0.001$). Data are from a single experiment; one independent experiment was performed.

Yong K et al. Supplementary Figure 5

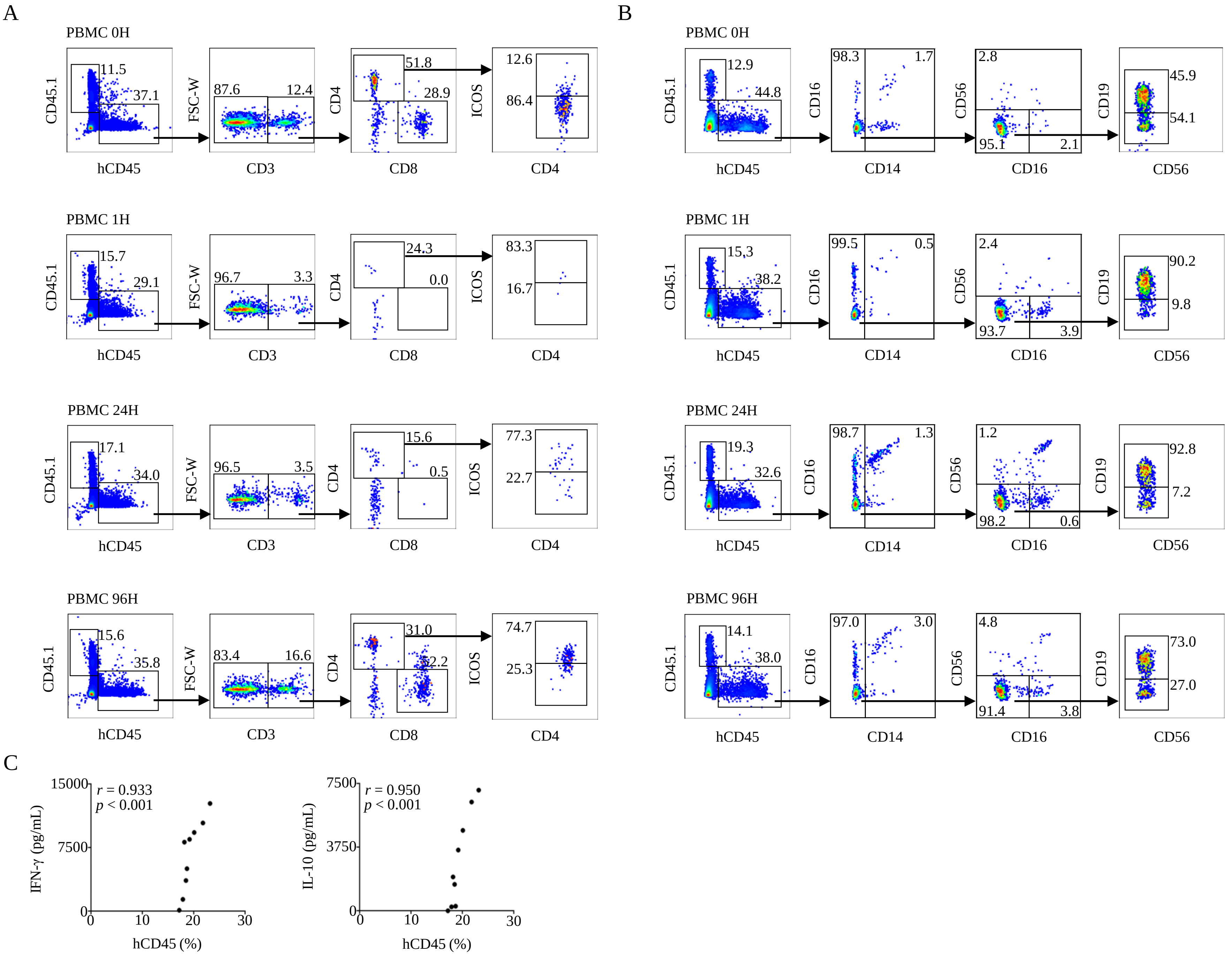

Supplementary Figure 5. Phenotype of cells from OKT3 treated humanized mice and correlation analysis between human reconstitution levels and cytokine production. (**A-B**) Immune profile of peripheral blood from humanized mice treated with OKT3. (**A**) Peripheral blood mononuclear cells (PBMCs) isolated from humanized mice were immunolabeled with mouse CD45.1, human CD45, CD4, CD8, ICOS and analyzed using flow cytometry. Representative flow cytometry plots illustrating the expression of $CD4^+$, $CD8^+$, $CD4^+ICOS^+$, $CD4^+ICOS^-$ for (**A**) PBMCs at 0H, 1H, 24H and 96H post-treatment are shown. (**B**) PBMCs isolated from humanized mice were stained for mouse CD45.1, human CD45, CD14, CD16, CD19, and CD56 and analyzed by flow cytometry. Representative flow cytometry plots showing the expression of monocytes, NK cells and B cells for (**B**) PBMC at 0H, 1H, 24H and 96H post-treatment are shown. The numbers illustrated are the frequency of the gated cells relative to the parent population. (**C**) Spearman's rank correlation coefficient analysis between the levels of $CD45^+$ cells to cytokines IFN-γ and IL-10 at 24H (n=10) after OKT3 administration.

Yong K et al. Supplementary Figure 6

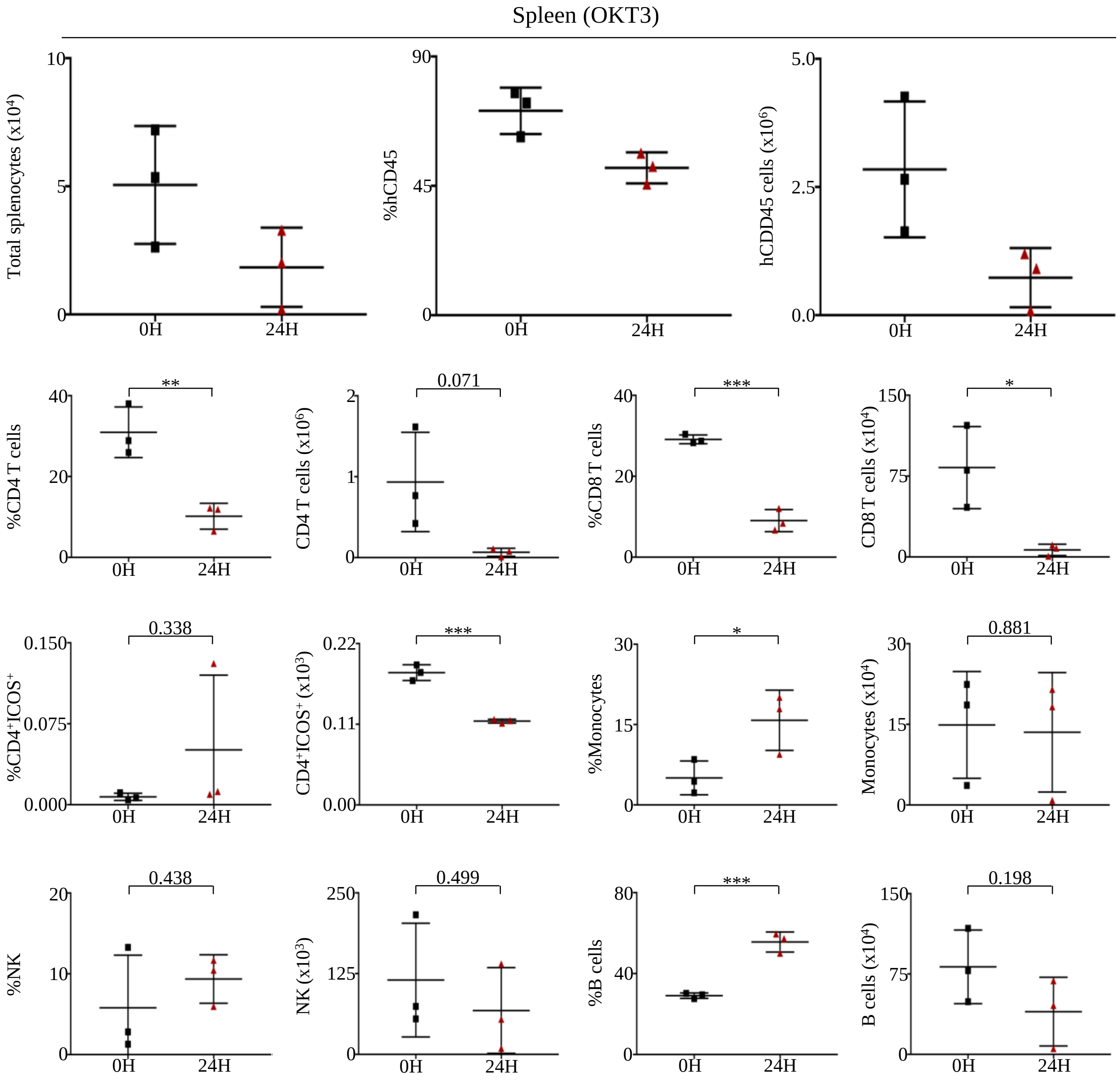

Supplementary Figure 6. Immune cell subset in spleen changes upon administration of OKT3. Immunophenotypic analysis of $CD4^+$ T cells, $CD8^+$ T cells, $CD4^+ICOS^+$ T cell subsets, NK cells, monocytes and B cell subsets within the spleen of OKT3 recipient humanized mice (n=3) at 0H and 24H post-treatment. Average absolute count and mean frequency of immune cells relative to total human $CD45^+$ cells ± SEM are shown. Two-tailed Mann Whitney *U* test; (*$p < 0.05$, **$p < 0.01$, ***$p < 0.001$). Data are from a single experiment; one independent experiment was performed.
