# Supplementary material for: Humanized Mouse as a Tool to Predict Immunotoxicity of Human Biologics
Source: Front Immunol. 2020 Oct 15;11:553362. doi: 10.3389/fimmu.2020.553362 (PMC7604536; doi:10.3389/fimmu.2020.553362)
Supplement: Supplementary Table 1 — Primers for qPCR. Primer sequences for FOXP3-TSDR demethylation-specific, FOXP3-TSDR methylation-specific, FOXP3, and β-actin. [file Table_1.pdf]

Yong K et al. Supplementary Table 1. Primers for qPCR. Primer sequences for FOXP3-TSDR demethylation-specific, FOXP3-TSDR methylation-specific, FOXP3 and β-actin.

Primer sets	Primer sequence	Product size	Cycle condition
FOXP3-TSDR demethylation-specific	Sense: TAGGGTAGTTAGTTTTTGGAATGA<br>Antisense: CCATTAACATCATAACAACCAAA	118 bp	Preheating: 98°C for 10 min; 40 cycles of 98°C for 15 seconds followed by 1 min at 60°C.
FOXP3-TSDR methylation-specific	Sense: CGATAGGGTAGTTAGTTTTCGGAAC<br>Antisense: CATTAACGTCATAACGACCGAA	113 bp	
FOXP3	Sense: TCCCAGAGTTCCTCCACAAC<br>Antisense: ATTGAGTGTCCGCTGCTTCT	122 bp	Preheating: 98°C for 10 min; 40 cycles of 98°C for 15 seconds followed by 1 min at 60°C.
β-actin	Sense: GGACTTCGAGCAAGAGATGG<br>Antisense: AGCACTGTGTTGGCGTACAG	234 bp	
